# Wanted: a better cut-off value for the Epworth Sleepiness Scale

**DOI:** 10.1007/s00508-017-1308-6

**Published:** 2018-01-16

**Authors:** Karin Trimmel, Magdalena Żebrowska, Marion Böck, Andrijana Stefanic, Daniel Mayer, Gerhard Klösch, Eduard Auff, Stefan Seidel

**Affiliations:** 1 Department of Neurology, Medical University of Vienna, Waehringer Guertel 18–20, 1090 Vienna, Austria; 2 Section for Medical Statistics, Centre for Medical Statistics, Informatics, and Intelligent Systems, Medical University of Vienna, Vienna, Austria

**Keywords:** Behavior rating scale, Narcolepsy, Predictive value of tests, Sleep latency, Hypersomnia

## Abstract

**Background:**

Excessive daytime sleepiness (EDS) is the main complaint in many neurological sleep disorders, such as idiopathic hypersomnia, narcolepsy, or obstructive sleep apnea/hypopnea syndrome (OSAS). The validity of the Epworth Sleepiness Scale (ESS) as a screening tool for EDS remains controversial. We therefore investigated (1) the interrelation of the ESS total score and the mean sleep latency (MSL) during the multiple sleep latency test (MSLT) and (2) the diagnostic accuracy of the ESS total score to detect EDS in patients with the chief complaint of subjective EDS.

**Methods:**

A total of 94 patients (48 males) with subjective EDS were included in this study. Regression analyses and ROC curve analyses were carried out to assess the predictive value of the ESS score for MSL.

**Results:**

The ESS score significantly predicted a shortened MSL (*p* = 0.01, β = −0.29). After dichotomizing into two groups, the ESS score predicted MSL only in patients with hypersomnia or narcolepsy (*p* = 0.01, β = −0.33), but not in patients with other clinical diagnoses (e. g. OSAS; *p* = 0.36, β = −0.15). The ROC curve analyses indicated an optimal ESS cut-off value of 16 with a sensitivity of 70%; however, specificity remained unsatisfactory (55.6%).

**Conclusions:**

Our results suggest that the predictive value of the ESS score in patients with subjective EDS is low and patient subgroup-specific (superior in hypersomnia/narcolepsy vs. other diagnoses) and that the commonly used cut-off of 11 points may be insufficient for clinical practice.

## Introduction

Excessive daytime sleepiness (EDS) is a key symptom of various neurological sleep disorders, such as idiopathic hypersomnia, narcolepsy, or periodic limb movement disorder [1–6]. Patients with obstructive sleep apnea syndrome (OSAS) frequently report EDS [7, 8]. The extent of daytime sleepiness may be quantified by the multiple sleep latency test (MSLT) and the Epworth Sleepiness Scale (ESS) score [9]. The MSLT is a validated objective measure for daytime sleepiness [10] and a sleep latency of ≤8 min is generally considered to indicate hypersomnia [3]. The MSLT requires the resources of a sleep laboratory and is time-consuming, which is obviously a limitation for its use as a screening method for hypersomnia. The ESS, an eight-item questionnaire to assess the extent of subjective daytime sleepiness, is easily administered and a cut-off of ≥10 points is accepted to indicate EDS [11].

The correlation between subjective and objective daytime sleepiness by comparison of ESS scores and mean sleep latency (MSL) during the MSLT has been the focus of a number of studies, but the results are conflicting [12, 13]. The vast majority of studies investigated the interrelation of ESS scores and MSL in patients suffering from OSAS [13, 14] and both a good [11, 15] as well as a limited [16, 17] predictive value of ESS scores for objective daytime sleepiness are reported. Although the ESS has been used in clinical studies on patients with narcolepsy, there is a lack of analyses of ESS scores and their relationship with MSL data from neurological sleep laboratories. Thus, we wanted to study the usefulness of ESS as a screening tool for daytime sleepiness in patients presented to our outpatient department for neurological sleep disorders. In detail, we investigated (1) the interrelation of the ESS score and the mean sleep latency (MSL) during the multiple sleep latency test (MSLT) and (2) the diagnostic accuracy of the ESS score to detect EDS in patients with the chief complaint of subjective EDS.

## Patients, material and methods

### Study sample and procedures

We reviewed the records of all consecutive patients at the outpatient department for sleep disorders of the Department of Neurology at the Medical University of Vienna who reported subjective EDS between January 2011 and May 2016. These patients had undergone a multiple sleep latency test (MSLT). Prior to the MSLT, patients had completed validated German versions of the ESS [11, 18] and the Pittsburgh Sleep Quality Index (PSQI [19]). To minimize possible confounding of MSLT results by preceding sleep deprivation [20], all subjects underwent polysomnography (PSG) the night before the MSLT. In the case of clinical suspicion, a sleep diary was administered and actigraphy was performed additionally in the week before the polysomnography and MSLT. Patients with insufficient sleep syndrome were not included in the study. Based on the medical history, the results of the MSLT and polysomnography, the final clinical diagnosis was established according to the 3rd edition of the International Classification of Sleep Disorders (ICSD-III [21]) by a sleep specialist (K. T or S. S.). Judgement on presence or absence of cataplexy was based on the patients’ clinical history. The study protocol was approved by the local Ethics Committee.

### Questionnaires

The ESS [11] is a self-rating instrument to evaluate the tendency to doze off during daytime. It consists of eight items concerning everyday situations. Responses to each item are ranked from 0 to 3 according to the probability for dozing off during a task (0 = never, 1 = low probability, 2 = moderate probability, 3 = high probability). A total score ≥10 indicates excessive daytime sleepiness. The PSQI [19] is a questionnaire that measures sleep quality over the previous month using 7 subscales measuring different components of sleep: subjective sleep quality, sleep latency, sleep duration, habitual sleep efficiency, sleep disturbances, use of sleep medication, and daytime dysfunction. Each component is reflected by a score ranging from 0 to 3, whereby 3 indicates a worse sleep quality. Good sleepers were defined as individuals with a PSQI score <5 and poor sleepers as individuals with a total PSQI score ≥5.

### Multiple sleep latency test (MSLT)

According to the latest practice parameters published by the American Academy of Sleep Medicine (AASM [22]), all patients underwent PSG in the night before the MSLT to ensure they had at least 6 h of nighttime sleep. A five nap MSLT was administered to all patients. All MSLT assessments were performed prior to the initiation of any behavioral (e. g. sleep hygiene training) or drug treatment (e. g. prescription of psychostimulants) related to the sleep complaints. Participants were asked to have 5 rests of 30 min every 2 h in a dark and quiet room with electroencephalogram (EEG; C3/A2, C4/A1, O1/A2, and O2/A1), electro-oculogram, and chin electromyogram. According to the standard practice of the American Sleep Disorders Association [10], sleep onset was determined visually by the first stage of sleep. If no sleep occurred, the trial was terminated and a sleep latency of 30 min [23] was recorded. The MSL was calculated as the mean of the five trials.

### Statistical analysis

Demographic data are presented as mean ± standard deviation (SD) or median + interquartile range (IQR) according to normal distribution of data. For group comparisons, unpaired t‑tests, Mann-Whitney U‑tests as well as χ^2^ tests were performed. Simple regression analyses were performed to examine the predictive value of the ESS total score (controlled for age and gender) for the objective measures of daytime sleepiness (MSL). Descriptive data in the text are reported as mean ± SD unless otherwise stated. Analyses were carried out for the whole cohort and after dichotomizing the cohort into patients with idiopathic hypersomnia/narcolepsy and patients with other sleep disorders. Furthermore, the receiver operating characteristic (ROC) curve was constructed and the area under the curve was calculated to identify the optimal cut-off value of the ESS to identify patients with objective EDS (i. e. MSL ≤8 min). The level of significance was set at 0.05. Statistical analyses were performed using SPSS v22.0 (SPSS Inc, Chicago, IL, USA) and Statistica v06 (StatSoft Inc., Tulsa, OK, USA).

## Results

The study sample included 94 patients (48 males) with an average age of 42 ± 16.3 years. The ESS and PSQI total scores of each patient were obtained 45 ± 43 days prior to MSLT. In this period, there was no behavioral (e. g. sleep hygiene training) or initiation of drug treatment (e. g. prescription of psychostimulants) related to the sleep complaints. Further demographic variables including number of sleep onset rapid eye movement (SOREM) episodes and PSQI scores are displayed in Table 1.

**Table 1:** Demographic characteristics. ESS, PSQI total scores, MSL and number of SOREM episodes, BMI, TST, NREM and REM of patients (*n* = 94) with subjective EDS

	*Mean*	*Min*	*Max*	*SD*
Age (years)	42.31	17	78	16.31
ESS	15.13	2	25	5.15
MSL (min)	8.85	0	20	4.77
TST (min)	364.0	241.5	492.5	71.2
NREM (% TST)	77.8	45.9	100.0	10.7
REM (% TST)	14.9	0.0	33.0	7.1
	*Median*	*Min*	*Max*	*IQR*
PSQI	7	0	17	4–10
SOREM	0	0	5	0–1
BMI	24.5	16.7	38.4	7.82

*BMI* body mass index, *ESS* Epworth Sleepiness Scale total score, *IQR* interquartile range, *MSL* mean sleep latency, *NREM* non-rapid eye movement sleep, *PSQI* Pittsburgh Sleep Quality Index, *REM* rapid eye movement sleep, *SD* standard deviation, *SOREM* number of sleep onset REM episodes, *TST* Total sleep time

The mean ESS total score was 15.1 ± 5.15 and the MSL was 8.85 ± 4.77 min. The majority of patients (56 patients, 59.6%) were diagnosed with idiopathic hypersomnia/narcolepsy, of which 30 patients (31.9%) were diagnosed with idiopathic hypersomnia, 17 patients (18.8%) with narcolepsy without cataplexy, and 9 patients (9.6%) had narcolepsy with cataplexy. Other diagnoses included OSAS (7 patients, 7.4%), insomnia (11 patients, 11.8%), somnambulism/somniloquy (4 patients, 4.3%), circadian sleep/wake-cycle disorder (4 patients, 4.3%), and restless legs syndrome/periodic limb movement disorder (RLS/PLMD, 4 patients, 4.3%). In 8 patients (8.6%) another primary neurological (migraine), internal (hypertension), or psychiatric (depression, dissociative disorder, anxiety disorder) condition was diagnosed. Of the patients 43% were taking medication relevant to their sleep complaints. Of these 15% were taking modafinil, 20% were taking selective serotonin reuptake inhibitors (SSRI), 9% were taking selective noradrenline reuptake inhibitors (SNRI), 4% were taking selective noradrenaline and dopamine reuptake inhibitors (SNDRI), 4% were taking tricyclic antidepressants (TCA), 4% were taking benzodiazepines, 5% were taking dopamine agonists, 3% were taking sodium oxybate, and 1% were taking atypical neuroleptics. Of all patients taking sleep-relevant medication, 81% were on monotherapy and 19% on dual or triple therapy. Comorbidities were present in 31% of patients and included general medical conditions (11%, hypertension, asthma, hypothyroidism), neurological conditions (9%, migraine, polyneuropathy, syncope), and psychiatric conditions (11%, depression, anxiety, personality disorder, history of substance abuse).

### Regression analysis

To investigate the relationship between the ESS and the MSL, a simple regression model was analyzed, where ESS was considered as a predictor. The analysis showed a significant negative linear dependence (*p* = 0.01; β = −0.29) between the MSL and ESS total score. To evaluate the influence of age, a simple regression model between the MSL and age was considered. Age was not significantly related to MSL (*p* = 0.88, β = 0.02). When regression analyses were restricted to patients with a diagnosis of idiopathic hypersomnia and narcolepsy (*n* = 56), the main result remained unchanged with a significant negative linear dependence for MSL (*p* = 0.01, β = −0.33). Once again, there was no effect of age (*p* = 0.13, β = 0.20). Regression analyses in patients with a diagnosis other than idiopathic hypersomnia/narcolepsy (*n* = 38) did not reveal a significant effect for MSL (*p* = 0.36, β = −0.15). The effect of age was also not significant (*p* = 0.18, β = −0.22). In order to control for possible confounding effects of gender, the study group was dichotomized into males (*n* = 48) and females (*n* = 46). The two gender groups were comparable with respect to ESS and PSQI total scores as well as MSL and number of SOREM episodes (Table 2). The distribution of diagnoses was comparable between males and females (Pearson χ^2^ = 0.16, Table 3), but mean age was lower in females compared to males (38.8 ± 14.4 vs. 45.7 ± 17.4 years; *p* = 0.04). Regression analyses were repeated for the two gender groups. In males, ESS showed a trend to predict MSL, but failed to reach statistical significance (*p* = 0.08, β = −0.26) and this effect was independent of age (*p* = 0.99, β = 0.002). In females, on the other hand, ESS significantly predicted MSL (*p* = 0.03, β = −0.31), which was also independent of age (*p* = 0.81, β = −0.04).

**Table 2:** Comparison of gender groups. Group comparisons (males vs. females) for variables ESS, MSL, SOREM, and PSQI. Unpaired t‑tests were performed for ESS and MSL and Mann-Whitney U‑tests were performed for SOREM and PSQI according to normal distribution of data

	Males (*n* = 48)		Females (*n* = 46)		
	*Mean*	*SD*	*Mean*	*SD*	*p (T)*
ESS	14.79	5.25	15.48	5.08	0.52
MSL	9.51	4.61	8.16	4.88	0.17
	*Median*	*IQR*	*Median*	*IQR*	*p (M-W)*
SOREM	0	0–2	0	0–1	0.50
PSQI	7	4–10	7	4–11	0.55

*ESS* Epworth Sleepiness Scale total score, *MSL* Mean Sleep Latency, *PSQI* Pittsburgh Sleep Quality Index, *SOREM* number of sleep onset REM episodes, *SD* standard deviation, *IQR* interquartile range, *p (T)* p-value according to unpaired t‑test, *p (M-W)* p-value according to Mann-Whitney U‑test

**Table 3:** Diagnoses and age groups in males vs. females

Diagnosis	*Males*		*Females*	
	*n*	Mean age (years)	*n*	Mean age (years)
Idiopathic hypersomnia	14	49.7	16	41.2
Narcolepsy without cataplexy	11	44.0	6	26.8
Narcolepsy with cataplexy	4	41.0	5	42.8
OSAS	6	61.7	1	58.0
Insomnia	5	47.6	6	44.0
Somnambulism/somniloquy	2	21.0	2	31.0
Circadian sleep/wake cycle disorder	1	40.0	3	22.0
RLS/PLMD	2	39.5	2	54.5
Other neurological/internal/psychiatric disorder	3	27.0	5	37.4

*RLS* restless legs syndrome, *PLMD* periodic limb movement disorder

**Table 4:** Linear regression analysis regarding the predictive value of single ESS items for MSL

	Activity	*p*	β
ESS item 1	Sitting and reading	0.05	−0.22
ESS item 2	Watching TV	0.06	−0.21
ESS item 3	Sitting inactive (public place)	0.22	−0.14
ESS item 4	Passenger in a car	0.61	−0.06
ESS item 5	Lying down to rest (afternoon)	0.91	−0.01
ESS item 6	Sitting and talking	0.21	−0.14
ESS item 7	Sitting after lunch	0.21	−0.14
ESS item 8	Car driver (stopped in traffic)	0.26	−0.13

*ESS* Epworth Sleepiness Scale, *MSL* mean sleep latency

### ROC analyses

**Fig. 1:**
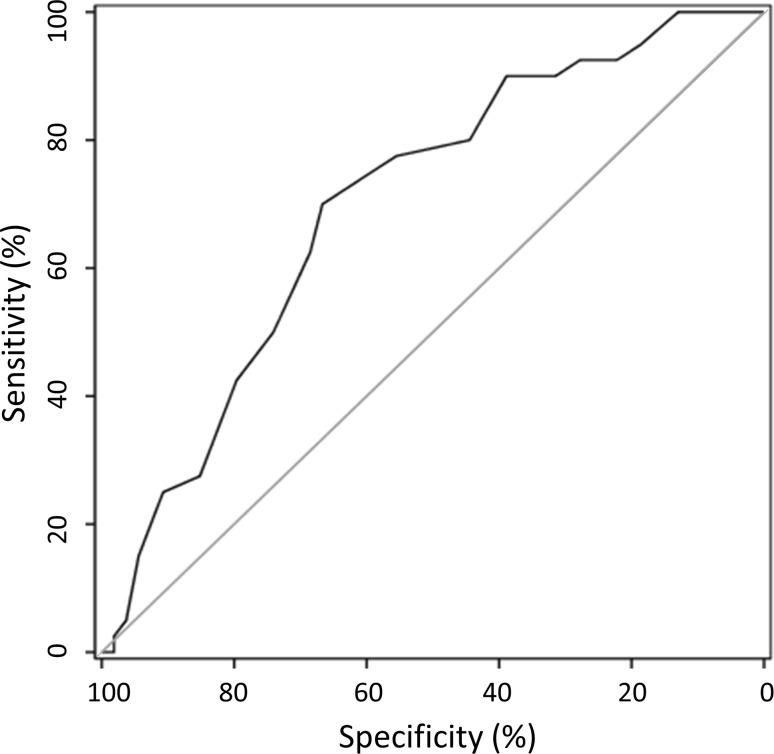
ROC curve of the ESS total score identifying patients with an MSL ≤8 min (*MSL* mean sleep latency)

**Fig. 2:**
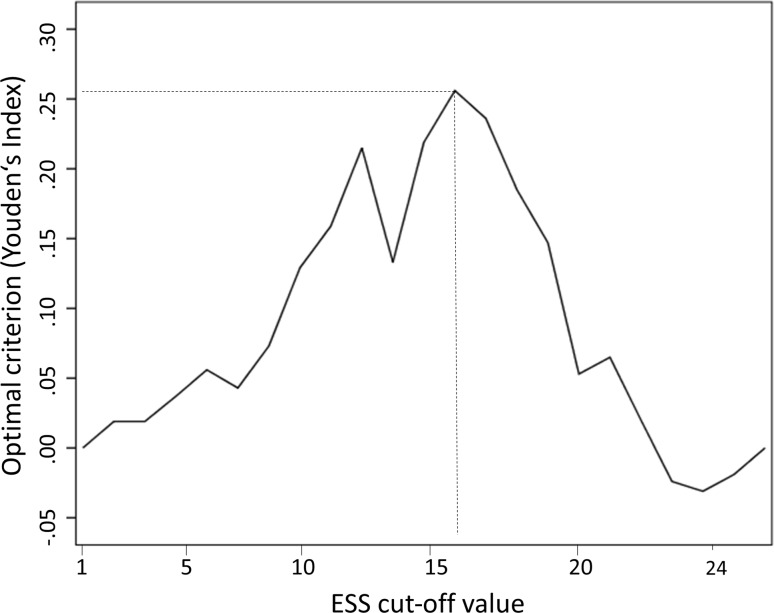
ESS cut-off values to predict an MSL ≤8 min related to the optimal criterion based on Youden’s Index (*MSL* mean sleep latency)

To evaluate the optimal cut-off value of the ESS score to detect excessive daytime sleepiness, an ROC curve analysis was performed with an MSL cut-off value of ≤8 min. The optimal cut-off value was selected by the criterion based on Youden’s Index [24, 25] defined as YI = max_c_ Se(c) + Sp(c) − 1, which maximizes sensitivity and specificity. The area under the ROC curve was 70.0% with a 95% CI between 59.4 and 80.6% (Fig. 1). The optimal ESS total score cut-off value was estimated to be 16, with a sensitivity of 70% and a specificity of 55.6% (Fig. 2) and a positive predictive value (PPV) of 61.7% and a negative predictive value (NPV) of 75%.

### Single-item analyses

It has previously been reported that scores of the 8 individual ESS items differ within subjects, which is attributed to the different grade of somnificity of the respective activity that is covered by each ESS question [26]. It has even been suggested that reduction to one single question might capture subjective daytime sleepiness comparably to the ESS [27]. In order to assess which of the 8 questions in the ESS best predicted a shortened MSL, regression analyses were performed for each ESS item. None of the single ESS items reached significance to predict the MSL. See Table 4 for details of the linear regression analysis.

## Discussion

In our study cohort with predominantly neurological sleep disorders (i. e. idiopathic hypersomnia or narcolepsy) we could show that the ESS score predicted a shortened MSL during the MSLT independent of age. Intriguingly, the predictive value of the ESS score only reached significance in female patients and patients with a diagnosis of idiopathic hypersomnia or narcolepsy.

Interestingly, the predictive value of the ESS scores for objective measures of sleepiness was gender-dependent, indicating a significant correlation of subjective and objective measures only in females, but not in males. It has previously been reported that women and men may report subjective sleepiness in different ways [28] and psychological factors, such as depression or anxiety, which have been shown to occur more frequently in women, can be associated with daytime sleepiness, but also that female gender per se can be an individual predictor for EDS [29]. This might be related to our findings, since there was a trend towards a shortened MSL in women.

The assessment of an optimal ESS score cut-off value using ROC analyses suggested a total ESS score of ≥16 to most likely identify patients with a pathologically shortened MSL, which is considerably higher than the generally accepted cut-off value of ≥10 points [11]. With the high cut-off value of ≥16 points, the sensitivity of the ESS score was 70% but specificity was only 55.6%. The respective PPV (61.7%) was considerably low, but the NPV was 75%. Our results are comparable to previous studies in patient populations primarily comprising patients with sleep-disordered breathing [30] although recently higher values of sensitivity/specificity for the ESS score have been reported in OSAS patients [14].

The utility of the ESS score in evaluating EDS and its relationship with objective sleepiness as expressed by the MSL are controversial topics. Previous studies reported an interrelation between ESS scores and MSL using simple correlation analyses [13, 30].; however, when using linear regression analyses [28] or survival analyses [13], no significant association was found. Therefore, the clinically accepted ESS score cut-off value of ≥10 points has been questioned with respect to its ability to predict a pathologically shortened MSL [30]. For instance, Aurora et al. [13] suggested a cut-off value of 13 to most effectively predict objective sleepiness in a study sample of which 48% were OSAS patients. Notably, most previous studies on the interrelation of subjective and objective sleepiness as expressed by ESS scores and MSLT results were primarily carried out in patients suffering from OSAS [13, 14, 17]. It has been described that ESS values tend to be higher in patients suffering from narcolepsy/hypersomnia as compared to OSAS [31] and one of the largest studies investigating the association of ESS and MSL [28] explicitly excluded patients suffering from narcolepsy or hypersomnia, assuming that the severity of sleepiness associated with these disorders might obscure any relation between the ESS score with the severity of sleep-related breathing disorders. Our study is strengthened by the fact that we analyzed a representative patient sample of a neurological sleep laboratory, which comprised a large portion of patients with subjective excessive daytime sleepiness caused by idiopathic hypersomnia or narcolepsy and we performed our regression analyses both for the entire cohort as well as after dichotomizing into two groups (narcolepsy/hypersomnia vs. other sleep disorders).

Since the diagnostic accuracy of the ESS as a screening tool for hypersomnia/narcolepsy has not been extensively studied in a non-trial setting and narcolepsy is still underrecognized or diagnosed with delay [32], our results provide novel and clinically relevant information on this topic because we investigated a cohort comprising a large proportion of patients suffering from hypersomnia/narcolepsy. Our findings suggest that a higher cut-off value of the ESS seems to be appropriate, but future studies with larger sample sizes, particularly for the comparison with other subgroups such as patients with sleep-related breathing disorders, are needed.

Limitations of the study include the modest sample size, especially concerning the subgroup analysis, as well as the lack of information on socioeconomic and employment status of subjects, since it has previously been shown that a lower degree of education can be associated with a higher predictive value of ESS for objective sleepiness [13]. It must be noted that due to technical reasons in our laboratory the MSLT was not carried out in complete accordance with the criteria proposed by Littner et al. [22], since nap trials where sleep did not occur were not terminated after 20 min, but 30 min, which has previously been validated in healthy subjects [23]. Previous studies report that PSQI and ESS scores might be only weakly associated with objective PSG measures, but are strongly related to psychological symptoms, in particular depression and anxiety [33–35]. Since ratings on symptoms of depression and anxiety were not collected in our study, a possible confounding of the results by psychological factors cannot be completely ruled out. It must be noted, however, that additional potential confounders like age and gender [28] were controlled for in our study and the number of individuals suffering from idiopathic hypersomnia/narcolepsy or other diagnoses was comparable between males and females. Females were slightly younger than males, but regression analyses did not indicate an influence of age on results. Another limitation is the lack of information on smoking status, since smoking has been suggested to be associated with an increased incidence of EDS [36]. All our patients were seen during clinical routine and thus, due to limited recording capacity in our sleep laboratory, questionnaire data were acquired prior to the performance of MSLTs and we were confronted with a certain lag between the ESS/PSQI and MSLT due to waiting times for the sleep studies (PSG and MSLT). In order to minimize therapeutic bias, we made sure that no therapeutic regimen was initiated in our patients prior to the MSLT. It must be noted that a relatively large proportion of patients were taking sleep relevant medication or were affected by comorbidities, which represents another potential confounding factor with an influence on ESS test results. Since our data were collected from patients at a tertiary centre, our findings cannot be generalized onto the population level.

In conclusion, the validity of the ESS to assess daytime sleepiness remains a topic of debate to this day and most validation studies arise from patients suffering from sleep-related breathing disorders, whereas neurological sleep disorders such as hypersomnia or narcolepsy are usually underrepresented. We investigated a cohort with a particularly large proportion of patients with hypersomnia or narcolepsy, which highlights the clinical significance of our findings for neurological sleep laboratories. We show that the ESS score predicts a shortened MSL, especially in patients with idiopathic hypersomnia or narcolepsy. An ESS total score of ≥16 points appears to predict objective EDS more reliably with modest sensitivity, although at the expense of a rather low specificity. Future studies in larger samples with specific subgroup analyses including gender effects are warranted to confirm and extend these findings.
